# Copper atomic-scale transistors

**DOI:** 10.3762/bjnano.8.57

**Published:** 2017-03-01

**Authors:** Fangqing Xie, Maryna N Kavalenka, Moritz Röger, Daniel Albrecht, Hendrik Hölscher, Jürgen Leuthold, Thomas Schimmel

**Affiliations:** 1 Institute of Applied Physics, Karlsruhe Institute of Technology, Campus South, 76128 Karlsruhe, Germany; 2 Institute of Microstructure Technology, Karlsruhe Institute of Technology, Campus North, 76021 Karlsruhe, Germany; 3 Institute of Electromagnetic Fields, ETH Zurich, 8092 Zurich, Switzerland; 4 Institute of Nanotechnology, Karlsruhe Institute of Technology, Campus North, 76021 Karlsruhe, Germany

**Keywords:** electrochemistry, encapsulation, metallic atomic-scale transistor, nanotechnology, photolithography

## Abstract

We investigated copper as a working material for metallic atomic-scale transistors and confirmed that copper atomic-scale transistors can be fabricated and operated electrochemically in a copper electrolyte (CuSO_4_ + H_2_SO_4_) in bi-distilled water under ambient conditions with three microelectrodes (source, drain and gate). The electrochemical switching-on potential of the atomic-scale transistor is below 350 mV, and the switching-off potential is between 0 and −170 mV. The switching-on current is above 1 μA, which is compatible with semiconductor transistor devices. Both sign and amplitude of the voltage applied across the source and drain electrodes (*U*_bias_) influence the switching rate of the transistor and the copper deposition on the electrodes, and correspondingly shift the electrochemical operation potential. The copper atomic-scale transistors can be switched using a function generator without a computer-controlled feedback switching mechanism. The copper atomic-scale transistors, with only one or two atoms at the narrowest constriction, were realized to switch between 0 and 1*G*_0_ (*G*_0_ = 2e^2^/h; with *e* being the electron charge, and *h* being Planck’s constant) or 2*G*_0_ by the function generator. The switching rate can reach up to 10 Hz. The copper atomic-scale transistor demonstrates volatile/non-volatile dual functionalities. Such an optimal merging of the logic with memory may open a perspective for processor-in-memory and logic-in-memory architectures, using copper as an alternative working material besides silver for fully metallic atomic-scale transistors.

## Introduction

Enormous research efforts worldwide are aimed at finding new solutions and technologies to manufacturing electronic components "beyond the silicon age". Resistive switching is one of the most promising alternative concepts. Resistive nanoelectronic devices are operated under one of the following switching mechanisms: electrostatic/electronic, electrochemical metallization, valence-change memory, thermochemical memory or phase change [1–5]. Resistive devices are a non-volatile and have been systematically investigated as two-terminal memristive devices with fast switching rate, high endurance, and long retention [6–8]. While two-terminal devices are able to constitute a completely new type of logic circuits as demonstrated by crossbar circuits [9], three-terminal devices can potentially be applied as logic operation devices that can fully utilize semiconductor circuit technology. Switching mechanisms such as valence-change memory effect, thermochemical memory effect, and electrochemical metallization effect have been previously exploited for three-terminal devices in solids [10–17]. But the performance of the solid resistive-switching three-terminal devices has to be improved in terms of switching rate, endurance and retention.

This work focuses on developing a three-terminal resistive device that utilizes the electrochemical metallization effect in an aqueous electrolyte to reduce mechanical stress during cycling. Silver atomic-scale transistors that operate in an aqueous nitric electrolyte at voltages in the millivolt range were previously demonstrated [18–22]. Here, we report our progress in the development of an atomic-scale transistor with a copper quantum point contact as switching block. The fabrication and electron-transport properties of metallic point contacts have been investigated both experimentally and theoretically [23–41]. The copper atomic-scale point contacts have been fabricated as mechanically controllable break junctions (MCBJ) [42–45], by using scanning probe microscopy (SPM) with a conductive tip [46–47], or electrochemical techniques [24–26,48–49]. Copper is an interconnection material in ultra-large scale integrated (ULSI) circuits, and its use as a working material for fully metallic atomic-scale transistors makes these devices adaptable for integration into advanced integrated circuits. The copper atomic-scale transistor is fabricated using standard photolithography and subsequent electrochemical deposition of the copper point-contact between two microfabricated gold electrodes (source and drain), and a thin copper film on a gate electrode. Its electrochemical operation parameters are explored systematically.

## Results and Discussion

**Figure 1:**
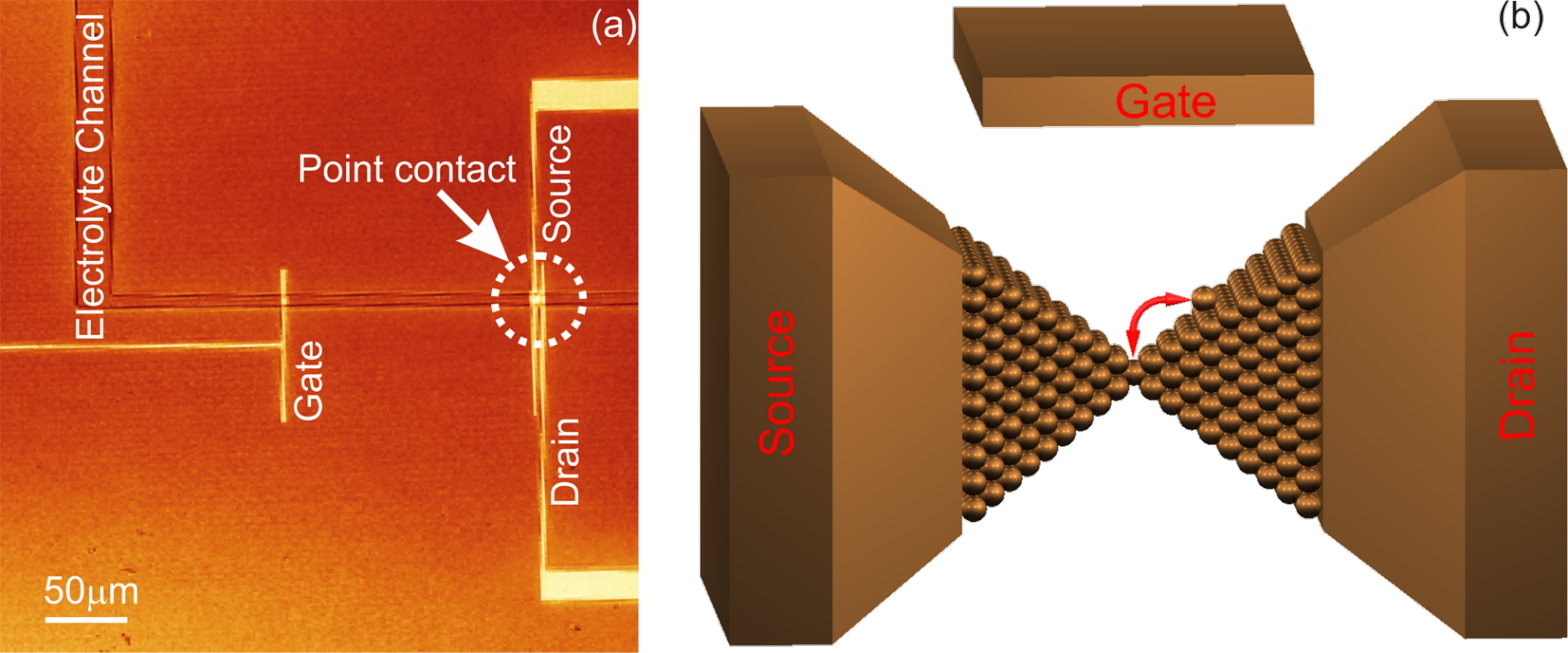
Copper atomic-scale transistor. (a) Confocal optical microscopy image of the microelectrodes, electrolyte channel and the copper point contact (indicated with a dotted circle), which was deposited between the source and the drain. (b) Schematic diagram of an atomic-scale transistor.

The fabrication of copper atomic-scale transistor consists of three steps. First, a silicon chip with three microfabricated electrodes – source, drain and gate – and a microchannel for on-chip electrolyte delivery are fabricated using standard photolithography (Figure 1a, Method 1 described in the Experimental section). The electrodes consist of Cr/Au films with a thickness of 3 nm/40 nm deposited on a silicon wafer covered with a thermal oxide layer (300 nm) using e-beam evaporation. The gap between the source and the drain is 2 μm, and the distance from the gate to the source is approximately 147 μm. The electrolyte channel developed in an SU-8 photoresist film is 8 µm wide and 2 μm high, as shown in Figure 1a.

In the second step, a copper film is electrochemically deposited on source and drain with a copper wire (99.9%, metal basis, diameter of 0.5 mm) as a counter electrode. During the deposition of the copper film source and drain become thicker and wider. Consequently, the gap between the two electrodes becomes narrower until, finally, a copper point contact is formed in the gap. The deposition continues for additional 20 min to stabilize the point contact. Simultaneously, a copper film is deposited on the gate electrode by grounding the gate electrode. After the stabilizing procedure, the gate electrode is disconnected from the ground and electrically suspended in the electrolyte in order to prevent copper dissolution from the gate during the following training procedure.

The third step is the training and switching procedure, which is similar to the one previously described for the silver atomic-scale transistors [21]. When the copper atomic-scale transistor can switch reproducibly, the copper wire as the counter electrode is disconnected electrically and taken out of the copper electrolyte. The gate electrode is reconnected as an electrochemical counter electrode. By changing the potential on the gate in a bipolar manner, the as-fabricated copper atomic-scale point contact is alternately dissolved and formed, resulting in resistive switching between the source and the drain. The sign and the magnitude of the voltage (*U*_bias_) applied across these two electrodes directly influence the growth of the quantum point contact. Because the electrochemical current is less than 1 nA in our experiment, the use of a reference electrode is not necessary. The conductance between the source and drain electrodes is measured with a current-to-voltage converter, in which the drain is virtually grounded via an operation amplifier. A bias voltage (*U*_bias_) is applied on the source electrode. Electrochemical deposition/dissolution is realized by changing the potential on the counter electrode. At the beginning of the experiment the electrochemical potential is set to 350 mV. A confocal optical microscopy image of the copper transistor is shown in Figure 1a. A schematic diagram of an atomic-scale transistor is illustrated in Figure 1b.

**Figure 2:**
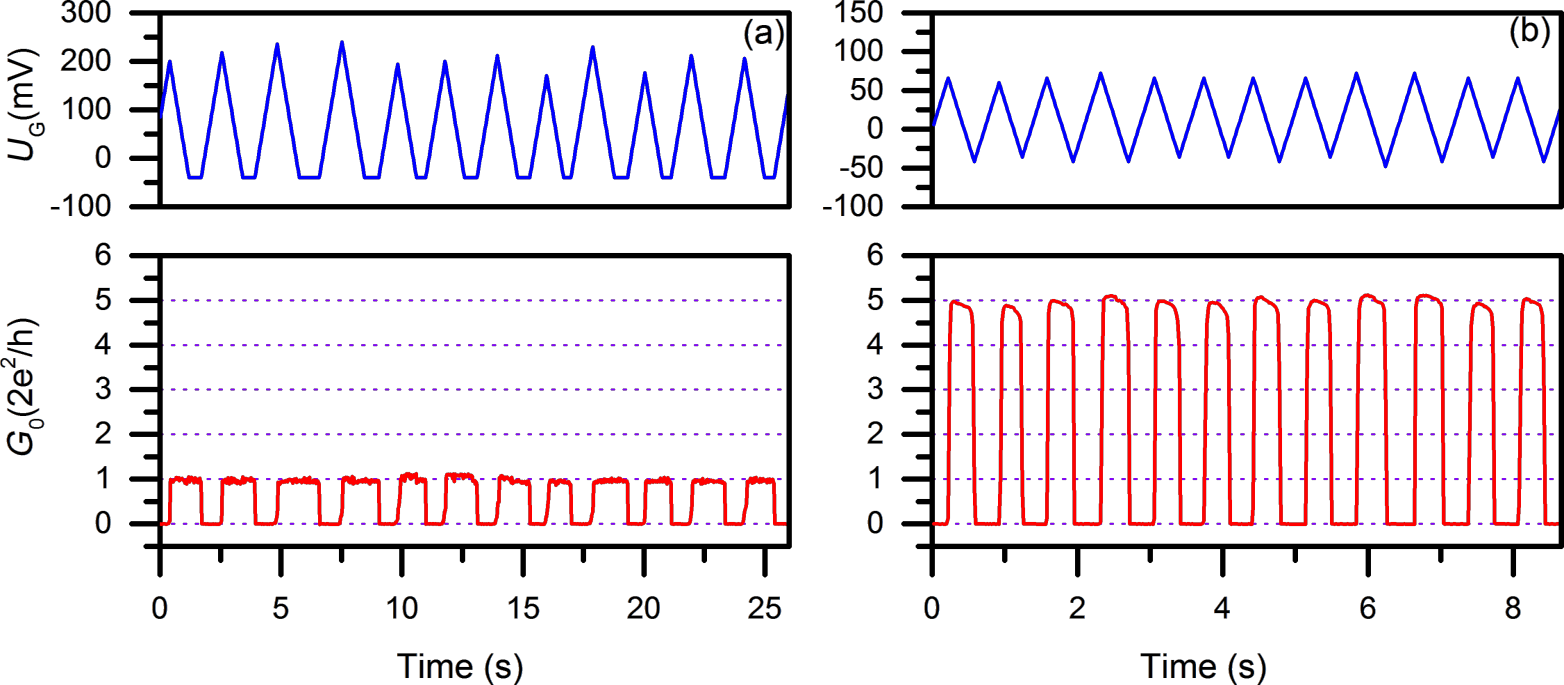
The performance of copper atomic-scale transistors. (a) 0–1*G*_0_ quantum conductance switching at 0.5 Hz. (b) 0–5*G*_0_ quantum conductance switching at 1.5 Hz.

To achieve conductance switching of copper atomic-scale transistors, the potential applied to the gate was controlled by a program developed in “NI LabVIEW” and the conductance was recorded simultaneously with the same program. The electrochemical potential was set using a feedback mechanism, in which the measured conductance was compared with a preset value of quantum conductance of the copper atomic-scale transistor. A transistor conductance switching between 0 and 1*G*_0_ (quantum conductance *G*_0_ = 2e^2^/h; with *e* being the electron charge, and *h* being Planck’s constant), and 0 and 5*G*_0_ results from the change of potential applied to the gate electrode (Figure 2). To operate the transistor, the chip is covered with a glass slide and sealed in the electrochemical cell. The electrolyte channel delivers the electrolyte from a reservoir to gate, source and drain. The electrolyte consists of copper sulfate, CuSO_4_ (5 mM), and sulfuric acid, H_2_SO_4_ (0.1 M), in bi-distilled water. The voltage applied between the source and drain electrodes is −12.9 mV in this case. With such a low applied voltage resistive switching is realized. The electrochemical potentials during operation are 220 mV (on) and −40 mV (off) for the switching between 0 and 1*G*_0_, and 72 mV (on) and −48 mV (off) for the switching between 0 and 5*G*_0_. The conductance switching between 0 and 1*G*_0_ demonstrates that only single copper atom is connecting source and drain.

**Figure 3:**
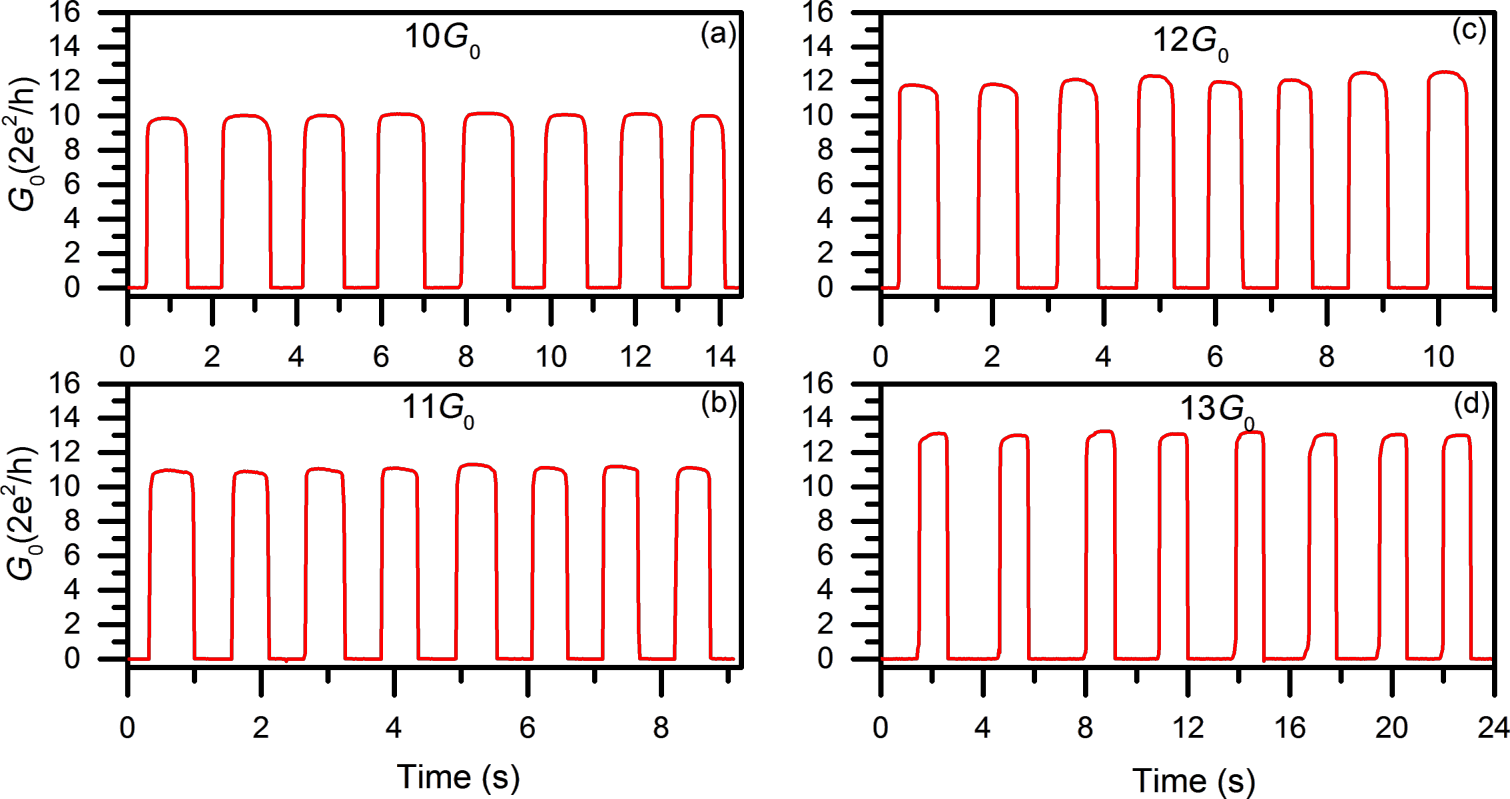
Controlled bistable quantum conductance switching of copper atomic-scale transistors. (a) 0–10*G*_0_ switching at 0.57 Hz, *U*_G_: −40 mV/210 mV (off/on). (b) 0–11*G*_0_ switching at 0.73 Hz, *U*_G_: −60 mV/120 mV. (c) 0–12*G*_0_ switching at 0.89 Hz, *U*_G_: −80 mV/140 mV. (d) 0–13*G*_0_ switching at 0.33 Hz, *U*_G_: −80 mV/240 mV.

To further investigate the performance of copper as a material for metallic atomic-scale transistors, copper atomic-scale transistors switching between 0 and arbitrary integer multiples of *G*_0_ were fabricated in which well-controlled, stable and long switching sequences could be achieved. Examples of the switching of copper atomic-scale transistors (0–10*G*_0_, 0–11*G*_0_, 0–12*G*_0_, and 0–13*G*_0_) are shown in Figure 3 with eight switching cycles presented for each case for better comparison.

**Figure 4:**
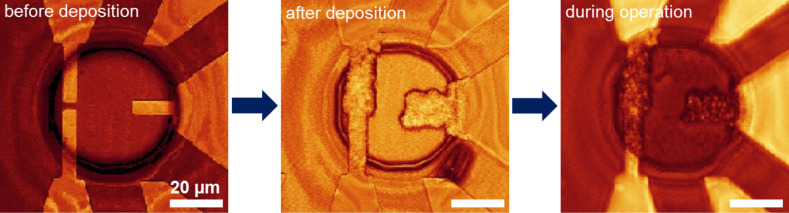
Observation of fabrication and operation of the copper atomic-scale transistor. Confocal microscopy images taken before electrochemical copper deposition, after deposition, and during transistor operation are shown (from left to right).

In order to observe fabrication and operation of the transistor in situ, and to test the operation of copper atomic-scale transistors in small volumes of electrolyte necessary for on-chip integration, chips with a circular window for containing the electrolyte were designed and fabricated with another standard lithography method using direct laser writing and reactive ion etching techniques (Method 2 described in the Experimental section). The window with a diameter of 3 mm and height of 0.05 mm was fabricated in the SU-8 photoresist. In order to observe fabrication and operation of the copper transistor in situ, a ceramic microscope objective was contacted with a drop of copper electrolyte (CuSO_4_ (4 mM) + H_2_SO_4_ (0.1 M)), and a copper wire serving as a counter electrode was partially immersed in the electrolyte. Electrochemical deposition/dissolution processes on the microelectrodes were observed in situ with a confocal microscope (Leica DMRXE, Germany). Images taken before the electrochemical deposition of copper, after the deposition, and during the operation of the copper atomic-scale transistor are shown in Figure 4. After initial copper deposition, the electrolyte on the chip was replaced with CuSO_4_ (10 mM) + H_2_SO_4_ (0.1 M), and a glass slide was placed on the top of the chip to encapsulate the electrolyte in the circular window. A higher concentration of CuSO_4_ was used for fabricating the point contact in the encapsulation. The insulating SU-8 layer is stable in the strong acidic electrolyte in which the devices are operated.

**Figure 5:**
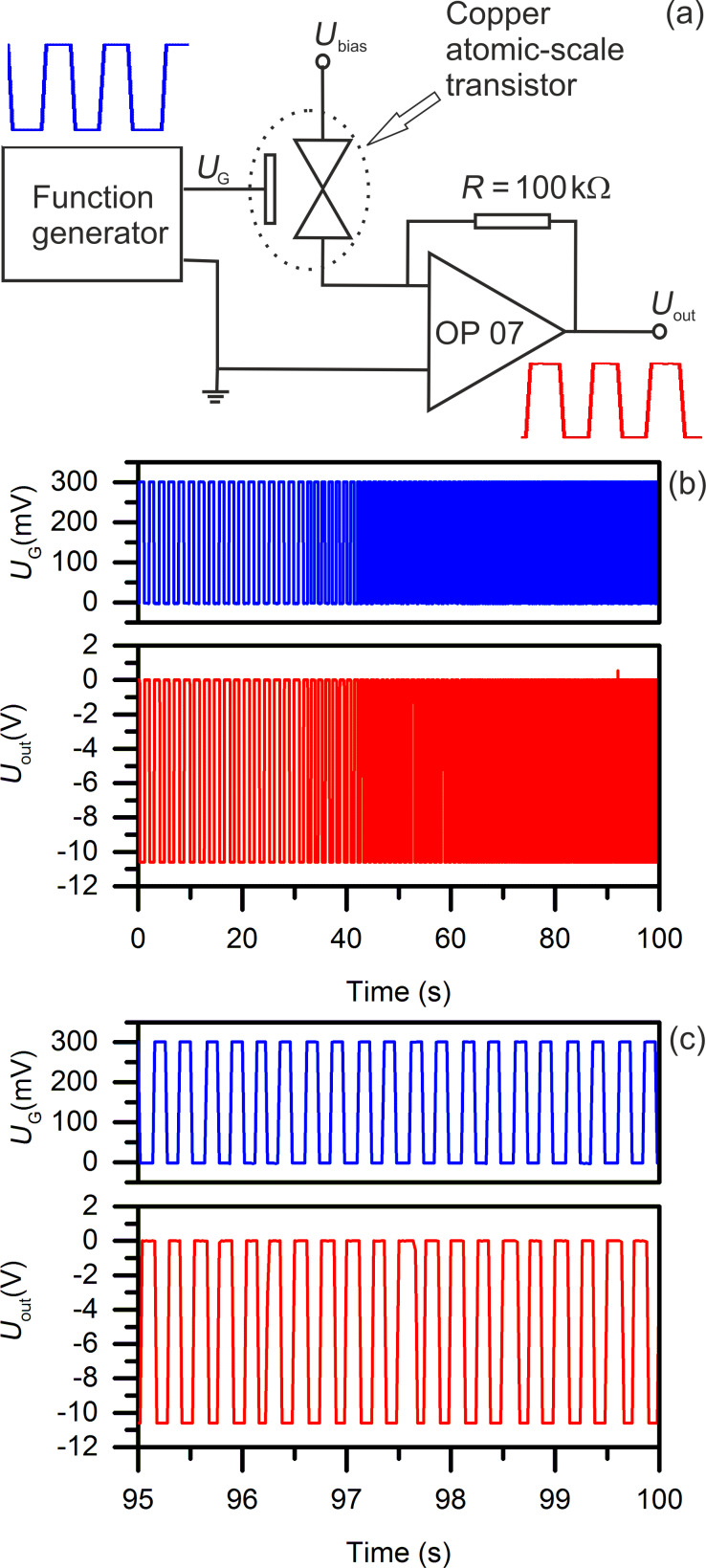
The operation of a copper atomic-scale transistor driven by a function generator. (a) Schematic diagram of the function-generator-driven copper atomic-scale transistor connected to a current-to-voltage converter circuit. (b) Rectangular wave signal from the function generator (blue) switched between 0 and 300 mV with frequencies from 0.5 Hz to 4 Hz. The output *U*_out_ (red), following the signal from the function generator, is driven between 0 and −10.6 V. (c) Zoom-in on the switching sequence from 95 to 100 s.

With the feedback mechanism the conductance switching is well controlled between “open” and “quantum conductance” (as displayed in Figure 2 and Figure 3), but the duration of each switching cycle varies. Moreover, the maximum/minimum potential values applied on the gate change from one cycle to another with a limit ramping rate of 300 mV/s. In order to get closer to real applications, the control of the device via a feedback mechanism was replaced with an external signal control. We demonstrate that the copper atomic-scale transistor can be operated with a signal from a function generator. The operation of the transistor driven with a function generator instead of the custom-developed software is demonstrated in Figure 5. The driving signal (rectangular wave) was applied by the function generator to the gate and switched the current between source and drain. Next, the current was converted to a voltage (*U*_out_) using the current–voltage converter circuit consisting of the copper point-contact, a resistor (*R*) and an operational amplifier (Analog Devices, OP07) (Figure 5a). The wide supply and input voltage ranges of the operational amplifier are ±15 V and ±14 V, respectively. The output signal *U*_out_ and the rectangular waveform generated with the function generator were simultaneously recorded using an A/D card (NI PCI-6221) with a custom-developed program. The maximum output of the A/D card is −10.6/10.6 V and the absolute read signal *U*_out_ is truncated by the A/D card when it exceeds 10.6 V. With *U*_bias_ = 120 mV), *R* = 100 kΩ, and *U*_out_ = −10.6 V, the quantum conductance of the copper point contact is calculated to be 11.4*G*_0_. Therefore, any quantum conductance higher than 11.4*G*_0_ is converted to −10.6 V with the setup illustrated in Figure 5a. The rectangular wave signal of the function generator switched between 0 and 300 mV with the frequency changing consecutively from 0.5 Hz to 4 Hz is shown in Figure 5b. The output signal *U*_out_ was driven between 0 and −10.6 V in the same manner. A zoom-in graph of the switching sequence between 95 and 100 s is shown in Figure 5c. The slope of the graph is an artifact due to the software sample rate (50 samples/s). All waveforms from the function generator as well as *U*_out_ are rectangular, as observed with an oscilloscope.

The magnitude of the bias voltage applied across source and has an important influence on the transistor switching rate. The switching rate obtained by setting *U*_bias_ at −12.9 mV is approximately 1 Hz. The sign and magnitude of *U*_bias_ change the electrochemical deposition behavior and shift the operation parameters of the electrochemical potential applied to the gate correspondingly. By setting *U*_bias_ at ca. 120 mV the switching rate can reach up to 10 Hz. With higher *U*_bias_ the copper atomic-scale transistor can be driven independently with the function generator instead of a computer.

**Figure 6:**
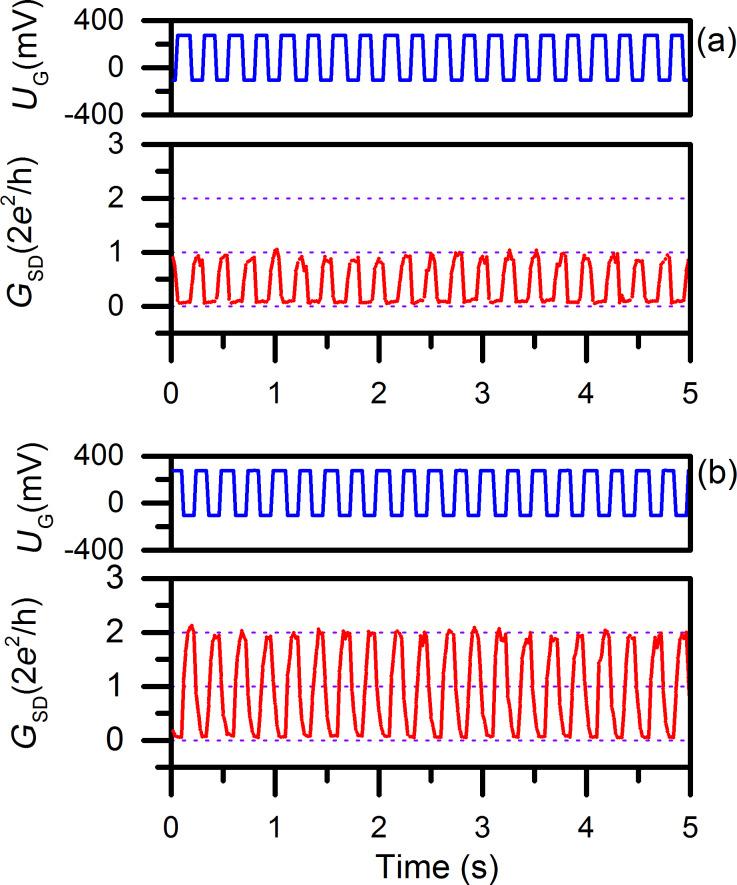
Demonstration of bistable quantum conductance switching of the copper atomic-scale transistor driven with a function generator. The bistable quantum conductance switching (red) between 0–1*G*_0_ (a) and 0–2*G*_0_ (b) follow the controlling rectangular wave (blue) at 4 Hz between −105 mV (off) and 275 mV (on) given by the function generator.

Using the electronic circuit described in Figure 5a, the quantum conductance of the atomic-scale transistor is measured with *U*_bias_ is set at 129 mV. Two switching sequences of quantum conductance between 0–1*G*_0_ and 0–2*G*_0_ are shown in Figure 6.

**Figure 7:**
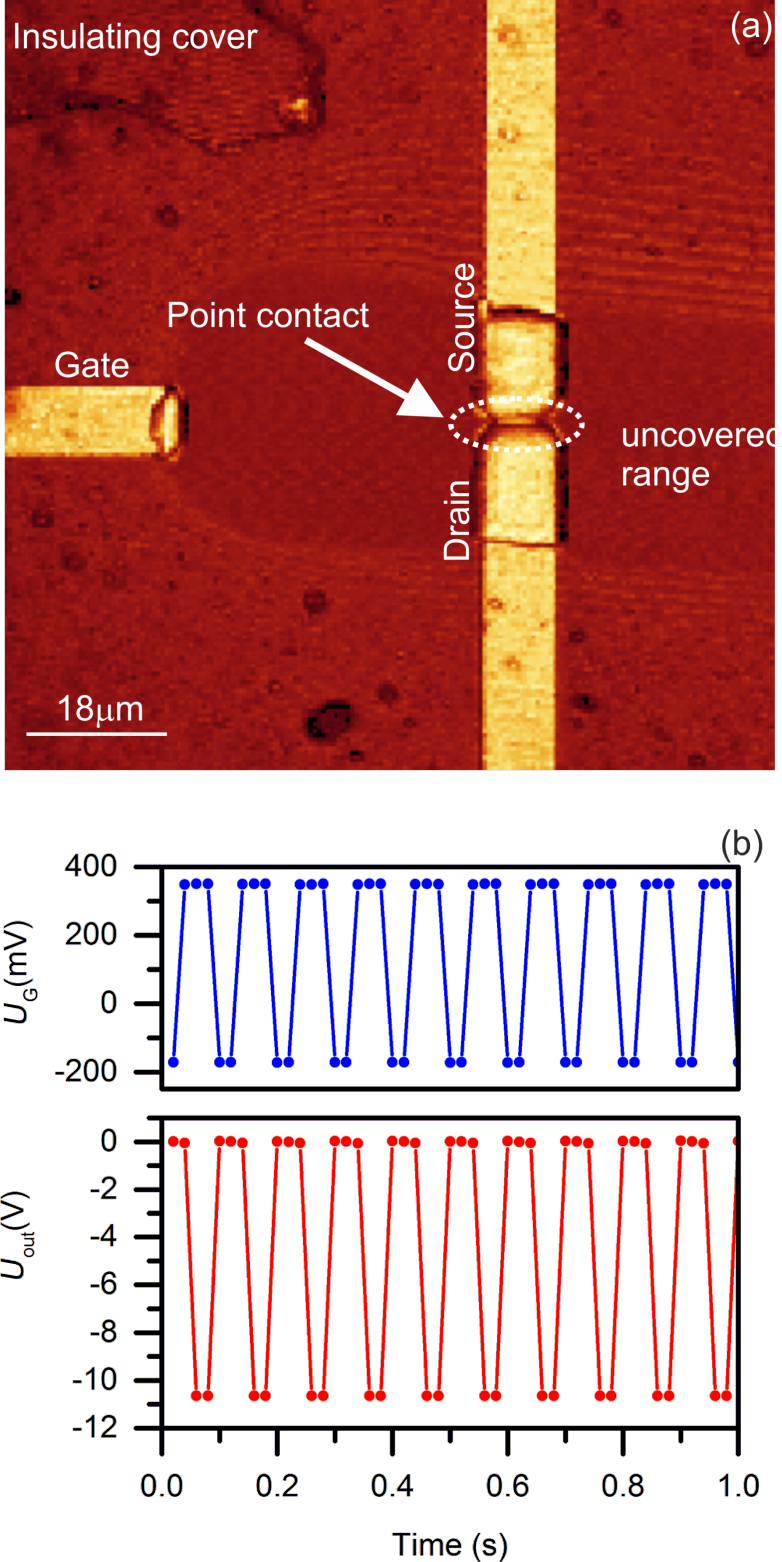
Influence of the copper electrolyte additives on the morphology of deposited copper film. (a) Confocal optical microscopy image of the copper atomic-scale transistor taken in situ. (b) The switching performance of the transistor implemented with a rectangular wave signal from a function generator applied to the gate at 10 Hz is plotted in the upper graph (blue). The output signal *U*_out_ is in the lower graph (red). The sampling rate of the recording software is 50 samples/s. The solid circles are the measured data and the lines are guides to the eye.

Copper electrolyte additives influence the morphology of the deposited copper film and, therefore, the switching behavior of the copper atomic-scale transistor. The copper electrolyte was prepared according to the recipe recommended by the supplier (Rohm and Haas, USA). The prepared electrolyte was diluted with bi-distilled water to 1:10 for better control over the electrochemical deposition/dissolution process during the fabrication of the copper atomic-scale transistor. The confocal microscopy image shown in Figure 7a was taken during the initial point-contact formation between source and drain. By comparing Figure 7a with Figure 4a we conclude, that the additives strongly influence the morphology of the deposited copper film and result in lower roughness. The sample used in this experiment was manually insulated with a polymer film. The switching sequence of the copper atomic-scale transistor driven by the function generator is displayed in Figure 7b with *U*_bias_ = 65 mV. As shown in Figure 7b, the rectangular wave signal switching between −170 and 350 mV at 10 Hz resulted in a rectangular *U*_out_ switching between 0 and −10.6 V.

In this work, the switching block of the copper atomic-scale transistor is a copper point contact. The dimension of its functional unit is comparable with a molecular electronic device. Within the molecular electronic functional building blocks, it is generally found that the electronic coupling between molecules and electrodes has a profound influence on the properties of the molecular devices [50–57]. Therefore, it is difficult to achieve the mass production of molecular electronic devices with identical configurations of their building blocks. Because of the difficult interface coupling between the molecular functional block and the two metallic electrodes, the whole resistance in the device is in the range of megaohms. The operation current of molecular electronics is in the range of nanoamperes, and the low operation current leads to a low signal-to-noise ratio. The interface-coupling problem between the functional block and the electrodes does not exist in the copper atomic-scale transistor, because the material of the electrodes and the building blocks is the same. The “switching on” current of a copper atomic-scale transistors is in the range of microamperes, which is compatible with modern semiconductor devices [58]. Moreover, the operational potentials of the copper atomic-scale transistor are in the range between −170 mV and 350 mV, which is in the magnitude range of the operation voltages (ca. 0.5 V) of the three most promising approaches, namely multigate transistors, tunnel field-effect transistors, and germanium nanodevices [59–62]. The dynamic power dissipation of the CMOS devices is proportional to the square of drain supply voltage (*V*_DD_) [58]. The copper atomic-scale transistors can be operated electrochemically with gate voltages less than 350 mV. This makes copper atomic-scale transistor a good alternative candidate for the development of electronic circuits with low power consumption.

## Conclusion

A copper atomic-scale transistor was fabricated on microelectrode chips in an electrolyte of copper sulfate and sulfuric acid in bi-distilled water. The operational potentials of the copper atomic-scale transistor are compatible with the operation voltages (ca. 0.5 V) of multigate transistors, tunnel field-effect transistors, and germanium nanodevices [59–62]. There is no interface coupling problem, which is common of molecular electronic devices, in the copper atomic-scale transistor, because the material of the electrodes and the functional building block based on a copper point-contact is metallic. The switching-on current of copper atomic-scale transistors is in the range of microamperes, which is compatible with modern semiconductor devices. The copper atomic-scale transistor can be encapsulated and operated in small electrolyte volume (3.5 µL). We demonstrate that the copper atomic-scale transistors operate as an independent electronic device controlled by a function generator, including also atomic-scale transistors with only one or two atoms in the narrowest constrictions. The switching rate reaches up to 10 Hz. All metallic atomic-scale transistors demonstrate volatile/nonvolatile dual functionalities and could be configured to perform both logic and memory operations. This merging of logic with memory opens perspectives for processor-in-memory and logic-in-memory architectures based on metallic atomic-scale transistors.

## Experimental

### Fabrication of microelectrodes

**Method 1:** Two masks with patterns for microelectrodes and windows in the insulating layer were prepared with a direct laser writer (Heidelberg Instruments DWL 66). The microelectrodes covered with an SU-8 film on a silicon wafer with a thin thermally oxidized SiO_2_ film (300 nm) were fabricated step by step as follows. A photoresist (AZ 5214E) was spin-coated on the wafer. With a mask aligner, the microelectrode pattern was transferred to the spin-coated photoresist. The photoresist film was developed in a developer (MIF 726). Cr/Au (3 nm/40 nm) films were evaporated with an e-beam evaporator (Lesker PVD75). The as-evaporated wafer was lifted in a photoresist remover in an ultrasonic bath. The wafer with the microelectrodes was spin-coated again with SU-8 to a thickness of 2 µm. The pattern with insulating windows was transferred to the spin-coated SU-8 film using the mask aligner. The wafer with the SU-8 film was developed in a developer (MicropositTM EC Solvent) and then baked at 210 °C for 1 h to solidify the SU-8 film.

**Method 2:** A photoresist (AZ 1505, 500 nm) was spin-coated on a thermally oxidized silicon wafer completely covered with evaporated Cr/Au (5 nm/60 nm) layer, and soft baked (95 °C, 2 min). The microelectrodes outline pattern was transferred into the photoresist film using direct laser writing (DWL 66, Heidelberg Instruments, Germany), and then developed. Reactive ion etching (RIE, Plasmalab100, Oxford Instruments, UK) was used to etch the Cr/Au film in the developed areas (120 W, 30 sccm Ar, 10 mTorr, 30 min) in order to isolate the microelectrodes from the surrounding Cr/Au film, and then the photoresist was stripped. To insulate the microelectrodes, an adhesion promoter (TI-Prime, MicroChemicals GmbH, Ulm, Germany) and SU-8 photoresist (3 µm) were spin-coated and soft baked (TI Prime: 120 °C, 2 min; SU-8: 95 °C, 5 min). The openings in the insulation layer were patterned using direct laser writing, and then developed after the post-exposure bake (PEB, 65 °C, 15 min). Next, the second SU-8 layer (50 µm) was spin-coated on the wafer, soft baked (95 °C, 20 min), exposed with direct laser writing, PEB (75 °C, 140 min) and developed. A circular window (*d* = 50 μm) above three microelectrodes and contact pad openings were patterned in the first insulating SU-8 layer (Figure 3). A larger circular window (*d* = 3 mm) (not shown in Figure 3) in the second SU-8 layer was used to contain the electrolyte for deposition and dissolution of the point contact.

### Fabrication of copper atomic-scale transistors

The copper atomic-scale transistor fabrication and operation experiments were performed in a Nylon electrochemical cell and observed in situ under a Leica confocal microscope. At the initial stage, a copper wire was employed as a counter electrode for copper deposition on the three microelectrodes source, drain and gate. After the gap between the source and drain electrodes was closed with the deposited copper the deposition process continued for about 20 min to stabilize the contact. The copper point contacts were trained as atomic-scale switching units. After these stabilization and training processes, the gate electrode was utilized as the counter electrode in the electrochemical deposition/dissolution cycling. Main components in the copper electrolyte were copper sulfate and sulfuric acid in bi-distilled water. Copper Gleam HS-200A, Copper Gleam HS-200 B (Rohm Hass, USA) and hydrochloric acid were added as additives to deposit uniform flat copper films. The copper electrolyte was prepared according to the recipe recommended by the supplier (Rohm Hass, USA): copper sulfate (CuSO_4_·5H_2_O), 100 g/L; sulfuric acid (96%), 200 g/L; chloride, 70 ppm (by adding hydrochloric acid); Copper Gleam HS-200A, 0.5 mL/L; Copper Gleam HS-200B, 10 mL/L.
